# The Impact of the COVID-19 Pandemic on Clinical Practice Education in South Korea: A Cross-Sectional Study

**DOI:** 10.3390/ijerph22040577

**Published:** 2025-04-07

**Authors:** Kuemju Park

**Affiliations:** Department of Nursing, The University of Suwon, Hwaseong-si, Gyeonggi-do 18323, Republic of Korea; longdr@suwon.ac.kr

**Keywords:** nursing education, clinical practice, COVID-19, nursing students

## Abstract

Background: This study aimed to investigate and analyze the remote teaching methods implemented in nursing education and clinical practice during the COVID-19 pandemic, with the aim of developing post-pandemic educational strategies. Methods: A structured questionnaire was administered to 92 full-time nursing professors nationwide, who were responsible for clinical courses in adult nursing. The data were analyzed using descriptive statistics via SPSS. Results: The majority (96.7%) of the participants implemented remote clinical practice during COVID-19. The primary teaching methods included virtual simulation programs (76.1%) and online nursing skills programs (75%). The clinical scenarios were primarily instructor-developed (63%) or drawn from virtual simulation cases (54.3%). Most professors (69.6%) modified their evaluation methods, and 85.9% reported changes in their assignment structures compared to their pre-pandemic practice. Conclusions: This study provides valuable insights into the effective remote clinical practice in nursing education, identifying specific programs and implementation considerations suitable for post-pandemic educational environments.

## 1. Introduction

### 1.1. Background

COVID-19 began to spread in 2020 and rapidly achieved worldwide coverage, with a high mortality rate, leading the World Health Organization to declare the outbreak a pandemic in March 2020 [1,2,3,4,5]. The COVID-19 pandemic led to the global normalization of non-face-to-face interactions, as governments worldwide implemented various lockdown measures, which significantly impacted mental and physical health, as well as education [6,7,8,9,10]. Following the Ministry of Education’s guidelines, universities began implementing remote education methods in earnest [11]. While pre-pandemic education was centered on instructors and knowledge transmission, in the post-pandemic period, education shifted towards learner-centered, competency-based, and problem-oriented approaches [12], highlighting the need for education that considers learners’ subjectivity [13]. Knowledge transfer was supplemented through online education systems [14,15,16], and nursing education also attempted to implement new teaching and learning methods using various media and materials in non-face-to-face settings [17,18,19].

Clinical practice in nursing education is an integrated type of course designed for students to apply their theoretical knowledge and skills in real-world settings [20]. The COVID-19 pandemic primarily affected clinical practice, where learning occurs through the direct and indirect observation and experience of nursing duties. As nursing students expressed fears about on-site clinical practice [21], most clinical training sought alternatives beyond hospital settings. Online nursing skills programs, which were utilized even before the pandemic, served as tools for nursing students’ skill learning, and virtual simulation programs were considered as alternative educational strategies for clinical practice, allowing students to safely experience nursing performance in virtual environments [22]. In non-face-to-face situations, efforts to implement competency-based clinical practice can be observed in nursing education through the use of small-group simulation practice and seminars within schools, securing face-to-face skill training time [23].

Research related to alternative clinical practice for nursing students includes studies on nursing students’ adaptation experiences through hospital clinical practice, even during the COVID-19 pandemic [21]; the exploration of nursing students’ experiences with alternative practices to replace hospital clinical practice [24,25]; and the confirmation of improvements in nursing students’ clinical performance and confidence [26,27,28], professionalism [29,30,31], and satisfaction [32]. However, it is difficult to find research on the specific non-face-to-face clinical practice methods developed by professors during the pandemic to replace existing clinical practice methods. From the instructor’s perspective, it is necessary to investigate specific aspects such as the evaluation methods adopted for changed clinical practice guidelines, tasks implemented to achieve learning outcomes, and changes in guidance for applying non-face-to-face clinical practice methods. It is also important to determine instructors’ thoughts about the application of non-face-to-face clinical practice methods so as to explore the various methods applicable in post-pandemic educational environments.

### 1.2. Aims

This study aimed to investigate and analyze alternative clinical practice methods. It was possible to obtain foundational data for the systematic organization of clinical practice course operation methods suitable for the changed educational paradigms and strategies implemented in the post-COVID-19 era.

The specific objectives of this study were as follows:

1. To identify the types of non-face-to-face clinical practice that replaced standard clinical practice during the COVID-19 pandemic;
2. To understand the operational methods of non-face-to-face clinical practice that replaced standard clinical practice during the COVID-19 period;
3. To examine instructors’ opinions on the use of the non-face-to-face clinical practice methods that replaced standard clinical practice during the COVID-19 period.

## 2. Research Methods

### 2.1. Research Design

This descriptive and cross-sectional study aimed to investigate the alternative forms and operations of clinical practice implemented during the COVID-19 period.

### 2.2. Research Subjects

The research subjects were 92 full-time faculty members, including assistant professors, associate professors, and professors, who were responsible for delivering adult nursing practice courses in nursing departments nationwide during the COVID-19 pandemic period, beginning in 2020. They were selected through convenience sampling, considering nationwide administrative districts. This study investigated the specific configurations of alternative practical education methods, student education programs, evaluation methods, and the opinions of nursing department professors who oversaw clinical practice during the pandemic period.

### 2.3. Research Tools

For this study, a survey questionnaire (Google Forms) was created to investigate the non-face-to-face practice methods that replaced standard clinical practice in nursing departments during the COVID-19 period. The survey content was developed by researchers who oversaw adult nursing courses and practical training, based on the clinical practice operation status during the COVID-19 period, according to the Korean Society of Adult Nursing in 2020 [12] and a literature review. The questionnaire was designed to align with the research objectives and comprised items regarding the operation of clinical practice, non-face-to face clinical practice methods, the content of non-face-to face clinical practice, programs utilized for non-face-to face clinical practice, and questions concerning instructors’ guidance methods and opinions. The questionnaire was developed in consultation with five nursing professors responsible for teaching adult nursing courses and clinical practice. It was designed considering the programs and practical operation methods used as alternatives to standard clinical practice, and its reliability and validity were verified.

### 2.4. Data Collection Method

Data collection was conducted through an online survey from 28 March to 20 April 2023. An explanation of the research was presented on the first page of the online survey, and the participants proceeded with the survey only after agreeing to participate. The data were collected by sending questionnaires, along with requests for research participation, to professors of adult nursing via email addresses obtained from nursing department websites listed on the Korean Nurses Association’s website. This method ensured the reliability and transparency of the data collection process.

### 2.5. Data Analysis Method

The collected data were analyzed using SPSS/WIN Statistics ver. 24.0. Subjects’ general characteristics and the content regarding non-face-to-face clinical practice and operations were analyzed in terms of frequencies and percentages. Descriptive responses to the semi-structured survey questions were analyzed using descriptive statistics.

### 2.6. Ethical Considerations for Research Subjects

This study was conducted according to standard operating procedures after receiving approval from the Institutional Review Board (The University of Suwon, IRB No. 2212-045-02). Participants, who voluntarily agreed to participate in this study, were informed about the research background and purpose, the research methods, the protection of their personal information and confidentiality, and their right to voluntarily refuse to participate or withdraw from the research at any time. Informed consent was obtained from all subjects involved in this study. Mobile gift certificates were provided to participating subjects as a token of appreciation.

## 3. Results

### 3.1. General Characteristics

The average educational experience of the research subjects amounted to 144.63 ± 95.16 months. The locations of the participants’ universities were distributed across Gyeonggi-do, Gangwon-do, Gyeongsangbuk-do, Chungcheongnam-do, and Chungcheongbuk-do and extended to Jeju-do. Overall, 69.6% of the subjects worked at schools without affiliated hospitals, while 30.4% worked at schools with affiliated hospitals (Table 1).

### 3.2. Non-Face-to-Face Clinical Practice Forms

Overall, 96.7% of the subjects had experience in replacing adult nursing clinical practice with non-face-to-face formats. In the first semester of 2023, 90.2% conducted clinical practice in hospitals in the pre-pandemic format, while 9.8% operated in mixed face-to-face/non-face-to-face practice. The duration of non-face-to-face clinical practice was most commonly one semester (34.8%), followed by two semesters (31.5%), three semesters (16.3%), and four semesters (14.1%). The reasons for substitution with non-face-to-face clinical practice included hospital requests (87%), student COVID-19 infections (40.2%), hospital COVID-19 patient infections (37%), university decisions (30.4%), and student requests (13%) (Table 2).

### 3.3. Alternative Clinical Practice Operation Methods

The methods used to replace clinical practice included online feedback (81.5%), virtual simulation programs (76.1%), online nursing skills programs (75%), self-study (72.8%), related video viewing (63%), self-produced video guidance (35.9%), clinical expert non-face-to-face lectures (14.1%), and electronic medical record (EMR) programs (14.1%). Conference methods were non-face-to-face (54.3%), face-to-face (15.2%), and mixed (30.4%). The cases used in practice were self-developed (63%), virtual simulation program cases (54.3%), existing practice cases (42.3%), and EMR cases (2.2%). Overall, 69.6% used evaluation methods that differed from their previous practices.

The alternative practice time was monitored through online program learning records (70.6%), assignment submission (67.4%), online conference attendance (55.4%), and online program completion certificates (46.7%). The assignment types differed from the pre-COVID-19 practice in 85.9% of cases, with 88% reporting an increased assignment volume. The practice guidance methods included online conferences (88%), online video guidance (68.5%), online feedback (66.3%), online lectures (42.4%), and face-to-face conferences (33.7%) (Table 3).

### 3.4. Detailed Program Operation

For online nursing skills programs, the allocation methods were autonomous learning (19.5%) and period-based allocation (89.5%). The performance verification methods included students’ submission of completion certificates and learning records (73.9%), as well as professors’ verification of learning records (20.3%). The utilization of virtual simulation programs included conferences (77.1%), case reports (41.4%), and self-study (21.4%). The verification of students’ performance on virtual simulation programs included performance score submission (97.1%) and professor-accessed record verification (50%). The core nursing skills practice methods included online nursing skills programs (53.3%), in-school practice (47.8%), video viewing (23.9%), online guidance (15.2%), and self-practice (3.3%). The core nursing skills evaluation methods included in-school face-to-face performance evaluations (56.5%), online oral evaluations (18.5%), skill performance video submissions (16.3%), written evaluations (15.2%), and online skill evaluations (8.7%) (Table 4).

### 3.5. Instructors’ Thoughts on Alternative Clinical Practice

The advantages of non-face-to-face clinical practice for students included time utilization (78.3%), physical comfort (62%), the utilization of various programs (48.9%), individualized guidance (28.3%), level-specific self-learning (27.2%), and stable training (19.6%). The disadvantages included the lack of direct observation (75%), the inability to verify communication (71.7%), the inability to use clinical equipment (50%), individual practice variations (28.3%), and the inability to experience clinical cases (28.3%).

For instructors, the advantages included the use of various programs (43.5%), ease of material use (38%), ease of practice guidance (35%), ease of individual feedback (23.9%), and in-depth conferences (10.9%). The disadvantages included the difficulty of designing the practice program (53.3%), evaluation difficulties (40%), case assignment difficulties (39.1%), difficulties evaluating core nursing skills (26.1%), and increased guidance time (7.6%). Overall, 73.9% of the subjects reported increased guidance time, while 21.7% reported no change. Regarding the achievement of learning outcomes through non-face-to-face clinical practice, 29.3% considered it appropriate, 47.8% moderate, 20.7% inappropriate, and 2.2% very inappropriate (Table 5).

## 4. Discussion

By discussing the results of this study and those of previous studies [23,33,34], which explored nursing students’ experiences with changed clinical practices and analyzed the effects of non-face-to-face practice, it is possible to obtain a three-dimensional understanding of the alternative clinical practices adopted during the COVID-19 pandemic period.

While this study found that 96.7% of the participants implemented alternative hospital practices during the COVID-19 pandemic period, research on nursing management operations during the same period showed that 69.2% conducted alternative hospital practices [35], revealing differences in the use of clinical practice alternatives between adult nursing and nursing management.

Online feedback was most commonly used as a non-face-to-face practice method, which aligns with the finding of individualized guidance being an advantage for students in non-face-to-face practice. This interpretation is consistent with previous research, where nursing students who experienced non-face-to-face clinical practice described individualized feedback as an advantage [33]. It suggests that online feedback provides advantages in terms of student-tailored feedback for both learners and instructors and could be considered for future clinical practice applications.

This aligns with research findings showing the use of virtual simulation programs and web-based technical training programs in medical and nursing student education during the same period [36]. Virtual simulation programs were used as cases for clinical practice, and their effectiveness in improving knowledge and problem-solving skills in clinical practice was confirmed [22]. International research showed that, during the COVID-19 pandemic period, computer-based simulation practice constituted 61%, and high-fidelity simulation constituted 12% of the alternatives to clinical practice [37], showing similar results to this study and suggesting the need to consider the advantages of related programs for clinical practice application in the post-pandemic era.

Self-study was used in 72.8% of cases as an alternative type of clinical practice, reflecting the difficulties faced by professors responsible for structuring the required clinical practice hours due to sudden hospital practice interruptions [35] and aligning with nursing students’ experiences, highlighting the lack of systematic organization in alternative practice [33]. However, self-study was considered as an alternative practice, as clinical practice inherently requires time for observation, self-reflection, and judgment [38]. Moreover, it should be noted that nursing students’ perceptions of self-study varied between positive and negative depending on their recognition types [25,39].

The use of self-produced videos and online clinical expert lectures as alternative methods reflects professors’ efforts to mimic clinical field experience and create alternative practice content. This is confirmed by research on the development of online clinical practice programs [40,41] and represents a consideration for future clinical practice applications. Clinical expert lectures were evaluated as efforts to complement the field presence of non-face-to-face clinical practice by presenting and explaining the clinical field environment online to nursing students who could not experience the hospital setting. This could be considered as an alternative for hybrid practice in the future.

Considering the unstable clinical practice due to unpredictable schedules that depended on the COVID-19 status and hospital field situation during the pandemic period [42], the provision of stable practice education through the use of various alternative programs was evaluated positively.

Clinical practice has longer completion hours compared to theoretical courses, so, when operating alternative clinical practices, nursing students’ participation time must be verified and reflected in their evaluations. For this, the overseeing professors directly verified their students’ program log records, confirmed their assignment submissions, checked their online conference attendance, and verified their online program certificates. This caused an increased workload compared to the existing clinical practice, and previous research has also pointed out the lack of preparation time for such professors [35], which is also reflected in the results of this study.

Online conferences and online feedback were common in specific guidance methods for alternative clinical practice, and previous research also showed that nursing students who experienced non-face-to-face practice viewed individual guidance and feedback positively, considering them advantages of this type of practice [38,40]. However, some cases reported insufficient instructor feedback during alternative practice [43], indicating the need for individual feedback to accompany online conferences and online feedback. This confirms the importance of educators’ feedback that takes nursing students’ practical experiences into account [44,45], as emphasized in traditional clinical practice education, highlighting the need for systematic evaluation and feedback [46].

Although standard clinical practice was reinstated after the pandemic, the use of online education programs and the shift to feedback-centered education is an ongoing trend experienced by both users and providers in clinical practice education. Based on the lessons learned from these trends, it is proposed that the application of non-face-to-face methods and the introduction of relevant programs be reflected in policies to enhance nursing education and facilitate adaptation to clinical practice in the future. This study identified the characteristics of alternative clinical practice from the instructor’s perspective, and future research suggestions include studying nursing students’ experiences with alternative clinical practice operations and conducting follow-up studies, such as qualitative meta-synthesis research, which could comprehensively verify the nursing students’ experiences with individual programs.

This study’s strength lies in its ability to conduct simultaneous online surveys with professors responsible for clinical nursing practice education across all regions of South Korea. This method ensured the reliability and transparency of the data collection process and allowed for a more detailed investigation into the provision of clinical practice education, enabling systematic analysis and the incorporation of participants’ opinions to accurately reflect the realities of non-face-to-face clinical practice education during the pandemic.

However, this study did have certain limitations. Although a convenience sample was drawn from across the nation, this study did not include aspects of clinical practice beyond adult nursing. Additionally, participants were selected using information from university websites, which may not have reflected recent changes, potentially introducing bias into the study results.

## 5. Conclusions

This study revealed that alternative clinical practice during the COVID-19 pandemic period was conducted using online programs, with changes in guidance methods, evaluation methods, assignment allocation, and assessment. For practical training operation that aligns with learning objectives in the rapidly changing post-COVID-19 environment, the overseeing professors could consider applying hybrid-type practices that incorporate field learning while developing systematic practice education content that does not depend on a physical hospital setting. This could be achieved by considering the advantages and disadvantages of the programs, guidance forms, assignment utilization, and conference operation methods used in alternative practice, as identified in this study.

Nonetheless, administrative and financial support from educational authorities is needed to develop clinical practice education programs, and policy support from relevant major academic societies and the Korean Accreditation Board of Nursing Education is deemed necessary. By systematically analyzing the results of research on the effectiveness of non-face-to-face clinical practice, alternatives to various forms of clinical practice education can be designed.

The significance of this research lies in its identification of the learning elements resulting from non-face-to-face clinical practice operations. Through this study, we can propose the application of and further research on the aspects of non-face-to-face education that can be utilized in nursing clinical practice.

## Figures and Tables

**Table 1:** General characteristics of participants (*n* = 92).

Variable	Category	*n* (%) or Mean ± SD
Gender	Female	88 (95.7)
	Male	4 (4.3)
Subject	Adult nursing	84 (91.3)
	Community nursing	3 (3.3)
	Administration nursing	3 (3.3)
	Fundamental nursing	2 (2.2)
Educational Career		144.63 ± 95.16 (months)
	1~10 years	43 (46.7)
	11~20 years	35 (38.0)
	21~30 years	10 (10.9)
	31 years~	4 (4.3)
Location	Seoul	8 (8.7)
	Busan	4 (4.3)
	Incheon	3 (3.3)
	Daegu	4 (4.3)
	Gwangju	2 (2.2)
	Daejeon	3 (3.3)
	Kyeonggi	12 (13.0)
	Gangwon	11 (12.0)
	Chungbuk	8 (8.7)
	Chungnam	9 (9.8)
	Gyeongbuk	10 (10.9)
	Gyeongnam	4 (4.3)
	Jeonbuk	8 (8.7)
	Jeonnam	5 (5.4)
	Jeju	1 (1.1)
Affiliated Hospital	Yes	28 (30.4)
	No	64 (69.6)

**Table 2:** Types of non-face-to-face practice (*n* = 92).

Variable	Category	*n* (%)
Type of practice		
2020~2022	Face-to-face	3 (3.3)
	Non-face-to-face (mixed)	89 (96.7)
2023	Face-to-face	83 (90.2)
	Non-face-to-face (mixed)	9 (9.8)
Duration of non-face-to-face practice	1 semester	32 (34.8)
	2 semesters	29 (31.5)
	3 semesters	15 (16.3)
	4 semesters	13 (14.1)
	5 semesters	3 (3.3)
Reason for non-face-to-face practice	Hospital’s opinion	80 (87.0)
	Students’ COVID-19 illness	37 (40.2)
	COVID-19 patients in hospital	34 (37.0)
	College decision	28 (30.4)
	Students’ opinion	12 (13.0)
	N.A.	3 (3.3)

**Table 3:** Operation of non-face-to-face practice (*n* = 92).

Variable	Category	*n* (%)
Type of replacement practice	Online feedback	75 (81.5)
	Virtual simulation	70 (76.1)
	Nursing skills online program	69 (75.0)
	Self-study	67 (72.8)
	Associated video program	58 (63.0)
	Instructor-created videos	33 (35.9)
	Clinical expert lectures (online)	13 (14.1)
	Electronic medical record (EMR) program	13 (14.1)
	Virtual reality (VR) program	4 (4.3)
	Augmented reality (AR) program	3 (3.3)
	In-school practice	1(1.1)
Type of conference	Face-to-face	14 (15.2)
	Non-face-to-face	50 (54.3)
	Mixed	28 (30.4)
Case for practice	Developed case	58 (63.0)
	Case of virtual simulation	50 (54.3)
	Existing practice case	39 (42.3)
	Case of electronic medical record (EMR)	2 (2.2)
Type of evaluation	Same as before	28 (30.4)
	Not same as before	64 (69.6)
Method used to record practice time	Log data for online program	65 (70.6)
	Submission of assignments	62 (67.4)
	Attendance of online conferences	51 (55.4)
	Certification for online program	43 (46.7)
Type of assignment	Same as before	13 (14.1)
	Not same as before	79 (85.9)
Amount of assignments	Increased assignments	81 (88.0)
	Decreased assignments	1 (1.1)
	No answer	10 (11.9)
Method of instruction	Online conference	81 (88.0)
	Online video	63 (68.5)
	Online feedback	61 (66.3)
	Online lecture	39 (42.4)
	Face-to-face conference	31 (33.7)

**Table 4:** Details of program operation.

Variable	Category		*n* (%)
Method of allocation of nursing skills program (*N* = 69)	Allocation	Weekly	16 (23.2)
		Daily	31 (44.9)
		Hourly	13 (18.8)
		Uncertain	2 (2.6)
	Self-discipline		15 (19.5)
Method used to check accomplishment of nursing skills (*N* = 69)	Students submit certification and learning records		51 (73.9)
	Teachers confirm log data		14 (20.3)
	Dual check (teacher and student)		23 (33.3)
Method of utilization of virtual simulation (vSim) (*N* = 70)	Conference		54 (77.1)
	Assignments for case report		29 (41.4)
	Self-discipline		15 (21.4)
	Running with teacher online		11 (15.7)
Method used to check accomplishment of virtual simulation (vSim) (*N* = 70)	Submit scores		68 (97.1)
	Check log data		35 (50.0)
	Reports		7 (10.0)
Method used to develop core nursing skills	Nursing skills program (online)		49 (53.3)
	In-school practice		44 (47.8)
	Authorized video		22 (23.9)
	Teaching online		14 (15.2)
	Self-discipline		3 (3.3)
Method of evaluation for core nursing skills	In-school skill test		52 (56.5)
	Online verbal test		17 (18.5)
	Skill performance video		15 (16.3)
	Written test		14 (15.2)
	Online skill test		8 (8.7)
	In-school verbal test		6 (6.5)
	Report		4 (4.3)
	Certification of nursing skills program		4 (4.3)
Replacement for written test	Test online		44 (47.8)
	Test in school		37 (40.2)
	No test		6 (6.5)
	Self-study		5 (5.4)
Replacement for learning outcomes regarding communication, cooperation among experts and professional ethics	Conference after assignment		36 (39.1)
	Utilization case		30 (32.6)
	Report		25 (27.2)
	Same as before		8 (8.7)
	Written test		2 (2.2)

**Table 5:** Opinions of instructors regarding alternative practice.

Variable	Category	*n* (%)
Advantages of non-face-to-face practice for students	Flexible use of time	72 (78.3)
	Physical comfort	57 (62.0)
	Utilization of various programs	45 (48.9)
	Individualized instruction	26 (28.3)
	Self-study by level	25 (27.2)
	Stable training	18 (19.6)
Disadvantages faced by students in non-face-to-face practice	Cannot observe nursing tasks directly	69 (75.0)
	Cannot experience clinical environment	66 (71.7)
	Cannot see communication	53 (57.6)
	Cannot experience clinical devices	46 (50.0)
	Individual practice deviations	26 (28.3)
	Cannot experience clinical cases	26 (28.3)
	Cannot experience cooperation among experts	24 (26.1)
	Cannot experience diagnostic study	14 (15.2)
	Cannot experience ethical issues	13 (14.1)
Advantages of non-face-to-face practice for teachers	Utilization of various programs	40 (43.5)
	Ease of use of data	35 (38.0)
	Ease of teaching practice	33 (35.0)
	Ease of individual feedback	22 (23.9)
	In-depth conferences	10 (10.9)
Disadvantages faced by teachers in non-face-to-face practice	Difficulty in organizing program	49 (53.3)
	Difficulty in evaluation	34 (40.0)
	Difficulty in allocation of cases	36 (39.1)
	Difficulty in evaluation of core nursing skills	24 (26.1)
	Increased instruction time burden	7 (7.6)
Time for instruction	Increased	68 (73.9)
	Same	20 (21.7)
	Decreased	4 (4.4)
Achievement of learning outcomes	Very appropriate	0 (0.0)
	Appropriate	27 (29.3)
	Typical	44 (47.8)
	Inappropriate	19 (20.7)
	Very inappropriate	2 (2.2)

## Data Availability

The data presented in this study are available on request from the corresponding author due to privacy.
